# TEMPORARY ACCOUNT HOLDS TO PROTECT VULNERABLE ADULTS AT RISK OF FINANCIAL EXPLOITATION

**DOI:** 10.1093/geroni/igad104.0884

**Published:** 2023-12-21

**Authors:** Marti DeLiema, Maruska Sommers, Carrie Binder

**Affiliations:** University of Minnesota, Minneapolis, Minnesota, United States; University of Minnesota School of Social Work, Saint Paul, Minnesota, United States; University of Minnesota School of Social Work, Saint Paul, Minnesota, United States

## Abstract

Elder financial exploitation presents a serious challenge to law enforcement and adult protective services (APS) agencies working to prevent losses. The goal of this project is to understand case characteristics associated with a new adult protection law in Minnesota–Chapter 45A–that authorizes temporary holds of suspicious transactions and withdrawal requests for up to 5 weeks to protect older and vulnerable adults experiencing exploitation. Most states have recently passed similar laws, but none have systematically collected data on when temporary holds are applied and for whom, what investigation takes place, and how interventions impact vulnerable and older adults’ financial safety. Analyzing administrative data on Chapter 45A, we found that 286 financial exploitation cases were referred for investigation and temporary holds in 2022 (mean= 23 cases per month) with approximately $3M in funds at risk of further losses. Temporary holds were implemented in 25% of the referred cases. More than 2/3rds of Chapter 45A cases involved fraud perpetrated by strangers compared to fewer cases of financial abuse by friends and family. There is substantial under-reporting of cases involving Black, Hispanic, American Indian, Asian/Asian Pacific Islander, and immigrant older and vulnerable adults relative to the size of these groups in Minnesota. More research is needed to determine why minority clients are not receiving equal access to Chapter 45A intervention, and how to remedy these disparities in case referrals. Greater outreach and education may help improve knowledge of the Chapter 45A reporting processes among financial institutions, law enforcement, and APS agencies that serve these populations.

